# High Prevalence of* Plasmodium falciparum* Infection in Asymptomatic Individuals from the Democratic Republic of the Congo

**DOI:** 10.1155/2016/5405802

**Published:** 2016-01-28

**Authors:** Dieudonné Makaba Mvumbi, Thierry Lengu Bobanga, Pierrette Melin, Patrick De Mol, Jean-Marie Ntumba Kayembe, Hippolyte Nani-Tuma Situakibanza, Georges Lelo Mvumbi, Célestin Ndosimao Nsibu, Solange Efundu Umesumbu, Marie-Pierre Hayette

**Affiliations:** ^1^Biochemistry and Molecular Biology Unit, Department of Basic Sciences, School of Medicine, University of Kinshasa, P.O. Box Kin XI, Kinshasa, Democratic Republic of the Congo; ^2^Department of Clinical Microbiology, University Hospital of Liege, 4000 Liege, Belgium; ^3^Department of Parasitology and Tropical Medicine, School of Medicine, University of Kinshasa, P.O. Box Kin XI, Kinshasa, Democratic Republic of the Congo; ^4^Department of Internal Medicine, School of Medicine, University of Kinshasa, P.O. Box Kin XI, Kinshasa, Democratic Republic of the Congo; ^5^Department of Pediatrics, School of Medicine, University of Kinshasa, P.O. Box Kin XI, Kinshasa, Democratic Republic of the Congo; ^6^National Malaria Control Program, P.O. Box Kin XI, Kinshasa, Democratic Republic of the Congo

## Abstract

Malaria remains a major public health problem in the Democratic Republic of Congo (DRC) with 14 million cases reported by the WHO Malaria Report in 2014. Asymptomatic malaria cases are known to be prevalent in endemic areas and are generally untreated, resulting in a significant source of gametocytes that may serve as reservoir of disease transmission. Considering that microscopy certainly underestimates the prevalence of* Plasmodium* infections within asymptomatic carriers and that PCR assays are currently recognized as the most sensitive methods for* Plasmodium* identification, this study was conducted to weigh the asymptomatic carriage in DRC by a molecular method. Six provinces were randomly selected for blood collection in which 80 to 100 individuals were included in the study. Five hundred and eighty blood samples were collected and molecular diagnosis was performed. Globally, almost half of the samples collected from asymptomatic individuals (280/580; 48.2%) had* Plasmodium* infections and the most species identified was* P. falciparum* alone in combination with* P. malariae*. The high prevalence reported here should interpellate the bodies involved in malaria control in DR Congo to take into account asymptomatic carriers in actions taken and consider asymptomatic malaria as a major hurdle for malaria elimination.

## 1. Introduction

Malaria is a parasitic disease caused by* Plasmodium* species transmitted to man by a mosquito bite [1]. Four* Plasmodium* species infect human but* Plasmodium falciparum* is responsible for the major morbimortality. Infection with* P. falciparum* can result in a simply asymptomatic carriage, uncomplicated or severe malaria. In fact, there is an exposure-related immunity in malaria that could explain these different expressions [2].

In high transmission settings, symptomatic malaria is often concerning children below five years as they have not been for long time exposed to the parasite and asymptomatic infections generally concern adults that acquired an antidisease and/or an antiparasite immunity during their exposure time [3–6]. Asymptomatic malaria cases are known to be prevalent in endemic areas [7–9] and are generally untreated, resulting in a significant source of gametocytes that may serve as reservoir of disease transmission [10].

The Democratic Republic of the Congo (DRC) is the second largest country in Africa and has a population that is estimated to be 75.5 million people. It is estimated that 97% of the population live in zones with stable transmission. In the WHO malaria report for 2014, 14 million of malaria cases were reported in DRC [11]. Some studies conducted in DRC revealed significant prevalence of asymptomatic* Plasmodium* carriers. Matangila et al. reported a prevalence of 21.6%, 27.4%, and 29.5% of asymptomatic* P. falciparum* infection in pregnant women in Kinshasa, respectively, by microscopy, Rapid Diagnostic Tests (RDTs), and PCR [12]. Maketa et al. found a prevalence of* Plasmodium* infection in asymptomatic children of 30.9% and 14.3% in two areas of Kinshasa, based on microscopic identification of thick blood smears [13]. The most recent available data are from a countrywide study that reports a prevalence of* Plasmodium* infection, in asymptomatic children less than five years, of 23% and 31%, respectively, determined by microscopy and RDT [14]. But Tiono et al. showed in their work that RDTs have some limits in detecting asymptomatic carriers of* P. falciparum* [15].

Currently, Polymerase Chain Reaction (PCR) assays are the most sensitive manner to detect* Plasmodium* DNA [16, 17] and it detects only viable* Plasmodium* [18]. Some PCR assays can detect less than 1 parasite/*μ*L as the technique validated by Cnops et al. [19].

Discrepancies have been often found between microscopy and PCR results for the determination of asymptomatic* Plasmodium* carriage. For example, Baliraine et al. reported 20.7% difference (12.6% by microscopy and 33.3% by PCR) [20], Dal-Bianco et al. found 25% difference (27% by microscopy and 52% by PCR) [21], and May et al. showed 19% difference (48.9% by microscopy and 67.9% by PCR) [22].

We conducted this study to assess the weight of asymptomatic carriage of* P. falciparum* in the DRC, based on a highly sensitive RT-PCR assay for malaria parasite identification.

## 2. Materials and Methods 

### 2.1. Study Sites and Participants

We randomly identified health areas within 6 provinces of the DRC with different malaria transmission dynamics: Bolenge in Equateur, Luzizila in Kinshasa, Mweka in Kasai-Occidental, Butembo in Nord-Kivu, Punia in Maniema, and Kapolowe in Katanga Province. This household survey recruited one hundred individuals per province, except for Maniema where only 80 individuals could be included. Every age and sex was taken into account after being given an informed consent by adults and parents/guardians of children. People with fever or other malaria related symptoms were not included in this study. Samples were collected during the period from March to November 2014.

### 2.2. Blood Collection and DNA Extraction

Five hundred and eighty blood samples were obtained from finger prick and dropped on filter paper (Whatman 3MM®) that were dried and stored into individual plastic bags with desiccant.* Plasmodium* DNA has been extracted using the QIAamp DNA Mini Kit® (Qiagen Benelux, Venlo, Netherlands) according to manufacturer's recommendation. Briefly, 3 circles of approximately 3 mm diameter were punched out from a blood spot and placed into a 1.5 mL microcentrifuge tube in which 180 *μ*L of buffer ATL was added. The final elution volume was 150 *µ*L. Each dried blood spot was treated individually into a sterile petri dish to avoid contamination. One negative control (sterile water) was included for each dozen of blood spots treated. DNA was stored at −20°C till further analysis.

### 2.3. Parasite Identification by Real-Time PCR

A real-time PCR having a sensitivity varying from 0.02 to 0.006 parasites/*μ*L for human* Plasmodium* species identification was run, as previously described by Cnops et al. [19]. PCR tests were run on a light cycler 480 instrument (Roche®) and in the presence of positive controls (provided by the Parasitology Unit, Institute of Tropical Medicine, Antwerp, and the Laboratory of Clinical Microbiology, University Hospital of Liège). PCR conditions were as follows: 2 min at 95°C, followed by 50 cycles of 15 s at 95°C and 60 s at 60°C.

### 2.4. Ethical Considerations

This study has received the ethical approbation of the Ministry of Public Health of the DRC and of the Institutional Committee of the Faculty of Medicine, University of Kinshasa.

## 3. Results and Discussion

*P. falciparum* was correctly identified in 280 samples (48.2%) among which 6 infections were mixed (*P. falciparum* +* P. malariae*). Prevalence of positive samples by age groups is represented in Table 1. The other human* Plasmodium* species* P. ovale* and* P. vivax* were not found. The real-time PCR cycles thresholds (Ct) vary from 21.72 to 39.23.

**Figure 1:**
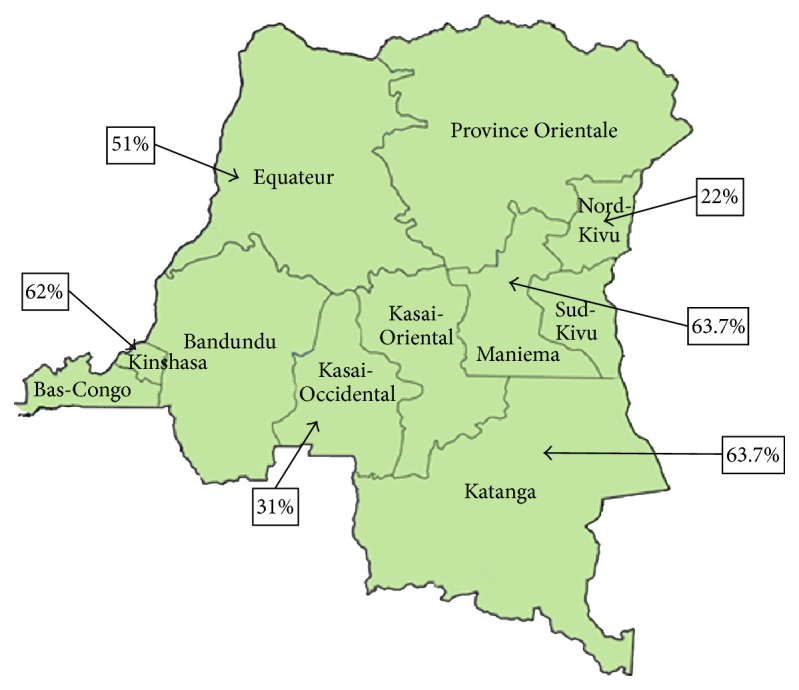
Prevalence of* P. falciparum* infection by collection sites based on a RT-PCR assay.

Prevalence of* P. falciparum* infection by province was 51% for Equateur, 62% for Kinshasa, 31% for Kasai-Occidental, 22% for Nord-Kivu, 63.7% for Maniema, and 63% for Katanga, as shown in Figure 1.

## 4. Conclusion 

Efforts to control or to eradicate malaria should take into account asymptomatic* Plasmodium* carriers because elimination of parasites in only symptomatic patients will not be enough as long as the pool of asymptomatic carriers will continue to act as a parasite reservoir [23]. Some researchers have suggested to screen and to treat asymptomatic carriers with an Artemisinin-based combination as part of a surveillance strategy towards malaria elimination [24, 25].

The high prevalence of* Plasmodium* infections in asymptomatic carriers found in this study stressed the importance of including this item in malaria control programs by the DRC Ministry of Health.

## Figures and Tables

**Table 1:** Prevalence of *Plasmodium* infections by age groups in the six DRC provinces.

Study sites	Age groups				Total
	0–5	6–15	16–59	>60	
	*N* (%)	*N* (%)	*N* (%)	*N* (%)	
Equateur	15 (29.4)	15 (29.4)	21 (41.1)	0 (0)	**51**
Kinshasa	7 (11.3)	10 (16.1)	45 (72.5)	0 (0)	**62**
K-occ	3 (9.6)	6 (19.3)	20 (39.2)	2 (6.4)	**31**
Kivu	2 (9.1)	5 (22.7)	15 (68.1)	0 (0)	**22**
Punia	4 (7.8)	6 (11.7)	37 (72.5)	4 (7.8)	**51**
Katanga	13 (20.6)	26 (41.2)	21 (33.3)	3 (4.7)	**63**

Total	**44 (15.7)**	**68 (24.2)**	**159 (56.7)**	**9 (3.2)**	**280**

K-occ = KasaÏ-occidental.
